# *Vibrio cholerae* Utilizes Direct sRNA Regulation in Expression of a Biofilm Matrix Protein

**DOI:** 10.1371/journal.pone.0101280

**Published:** 2014-07-23

**Authors:** Tianyan Song, Dharmesh Sabharwal, Jyoti Mohan Gurung, Andrew T. Cheng, Annika E. Sjöström, Fitnat H. Yildiz, Bernt Eric Uhlin, Sun Nyunt Wai

**Affiliations:** 1 Department of Molecular Biology, Umeå University, Umeå, Sweden; 2 The Laboratory for Molecular Infection Medicine Sweden (MIMS), Umeå University, Umeå, Sweden; 3 Umeå Centre for Microbial Research (UCMR), Umeå University, Umeå, Sweden; 4 Department of Microbiology and Environmental Toxicology, University of California Santa Cruz, Santa Cruz, California, United States of America; University of Würzburg, Germany

## Abstract

*Vibrio cholerae* biofilms contain exopolysaccharide and three matrix proteins RbmA, RbmC and Bap1. While much is known about exopolysaccharide regulation, little is known about the mechanisms by which the matrix protein components of biofilms are regulated. VrrA is a conserved, 140-nt sRNA of *V. cholerae*, whose expression is controlled by sigma factor σ^E^. In this study, we demonstrate that VrrA negatively regulates *rbmC* translation by pairing to the 5′ untranslated region of the *rbmC* transcript and that this regulation is not stringently dependent on the RNA chaperone protein Hfq. These results point to VrrA as a molecular link between the σ^E^-regulon and biofilm formation in *V. cholerae*. In addition, VrrA represents the first example of direct regulation of sRNA on biofilm matrix component, by-passing global master regulators.

## Introduction

*Vibrio cholerae* inhabits aquatic environments and when it enters the human intestine, e. g., through ingestion of contaminated food or water, it causes the severe diarrheal disease, cholera. Vibrios are shown to form biofilms on zooplankton, insects and intestines [1]–[5]. Compared to planktonic cells, bacteria within biofilms are more resistant to stress conditions, e. g., osmotic and oxidative stress, acidity, antibiotics exposure and immune clearance [6]–[12]. Biofilm structures are constructed of and maintained by biofilm matrix components [13]. In *V. cholerae*, formation of biofilm requires production of exopolysaccharide (VPS) and the biofilm matrix proteins RbmA, RbmC and Bap1 [14]–[18]. These matrix proteins appear to be involved at particular steps during the biofilm formation process. RbmA is involved in the initial cell-cell adhesion step and serves as a tether, forming flexible linkages between cells and the extracellular matrix [18], [19]; Bap1 facilitates adherence of the developing biofilm to surfaces; and the heterogeneous mixtures of VPS, RbmC and Bap1 appear to form envelopes to encase the cell clusters [18]. Without RbmC, incorporation of VPS through the biofilms is significantly reduced, suggesting an essential role for RbmC in maintaining the mature biofilm structure [18].

To date, studies on the regulation of biofilm formation have been mainly focused on VPS synthesis. A complex regulatory network controls transcription of the *vps* gene in response to multiple environmental signals, such as signals from quorum-sensing bacterial autoinducers [20], polyamines [21], [22], nucleosides [23], [24], indole [25] and nutrient scarcity [26]. Recently, glucose-specific enzyme IIA has also been shown to regulate biofilm formation through binding to a carbon storage regulator homolog MshH, demonstrating a link between the phosphoenolpyruvate phosphotransferase system and biofilm formation [27], [28]. In contrast to the vast body of knowledge about VPS regulation, very little is known about regulation of the matrix proteins (RbmA, RbmC and Bap1). Fong et al [29] has demonstrated the involvement of two factors: the cyclic AMP (cAMP)-cAMP receptor protein (CRP) complex and a transcriptional regulator VpsR. While VpsR positively regulates transcription of the *rbm* genes, cAMP-CRP appears to negatively regulate *rbm* expression, both mediated by and independently of VpsR [29].

In the past decade, an increasing body of evidence has highlighted the important and complex roles of small regulatory RNAs (sRNAs) in bacterial physiology and pathogenesis [30], [31]. Many sRNAs are produced in response to specific environmental signals/stresses. They act by base-pairing with target sequences, resulting in up- or down-regulating gene expression through modulating the translation or the turnover of target mRNAs (see review [32]). This mechanism of regulation often requires the RNA chaperone protein Hfq that facilitates base pairing between sRNAs and their target mRNAs [33], [34]. In *Vibrio*, a σ^E^-dependent sRNA, VrrA, has been shown to be induced by envelope stress and to repress the outer membrane proteins OmpA and OmpT through base pairing to the 5′ untranslated regions (UTR) of the corresponding mRNAs. When the OmpA level decreases, envelope stress is reduced by releasing outer membrane vesicles (OMVs) [35], [36]. These OMVs further protect bacteria against environmental hazards such as UV damage [37]. Using the infant mouse model, VrrA was demonstrated to attenuate *V. cholerae* virulence [35], which could be partially explained by the VrrA-mediated down-regulation of TcpA, a major *V. cholerae* virulence factor essential for host colonization. In this study, we provide evidence that VrrA down-regulates the biofilm matrix protein RbmC by base-pairing with the 5′-UTR of *rbmC* mRNA. Because RbmC is essential for maintaining the mature structure of biofilms, this VrrA-mediated suppression of RbmC might be an additional mechanism of biofilm regulation in *V. cholerae*.

## Results

### VrrA down-regulates RbmC independently of Hfq

**Figure 1:**
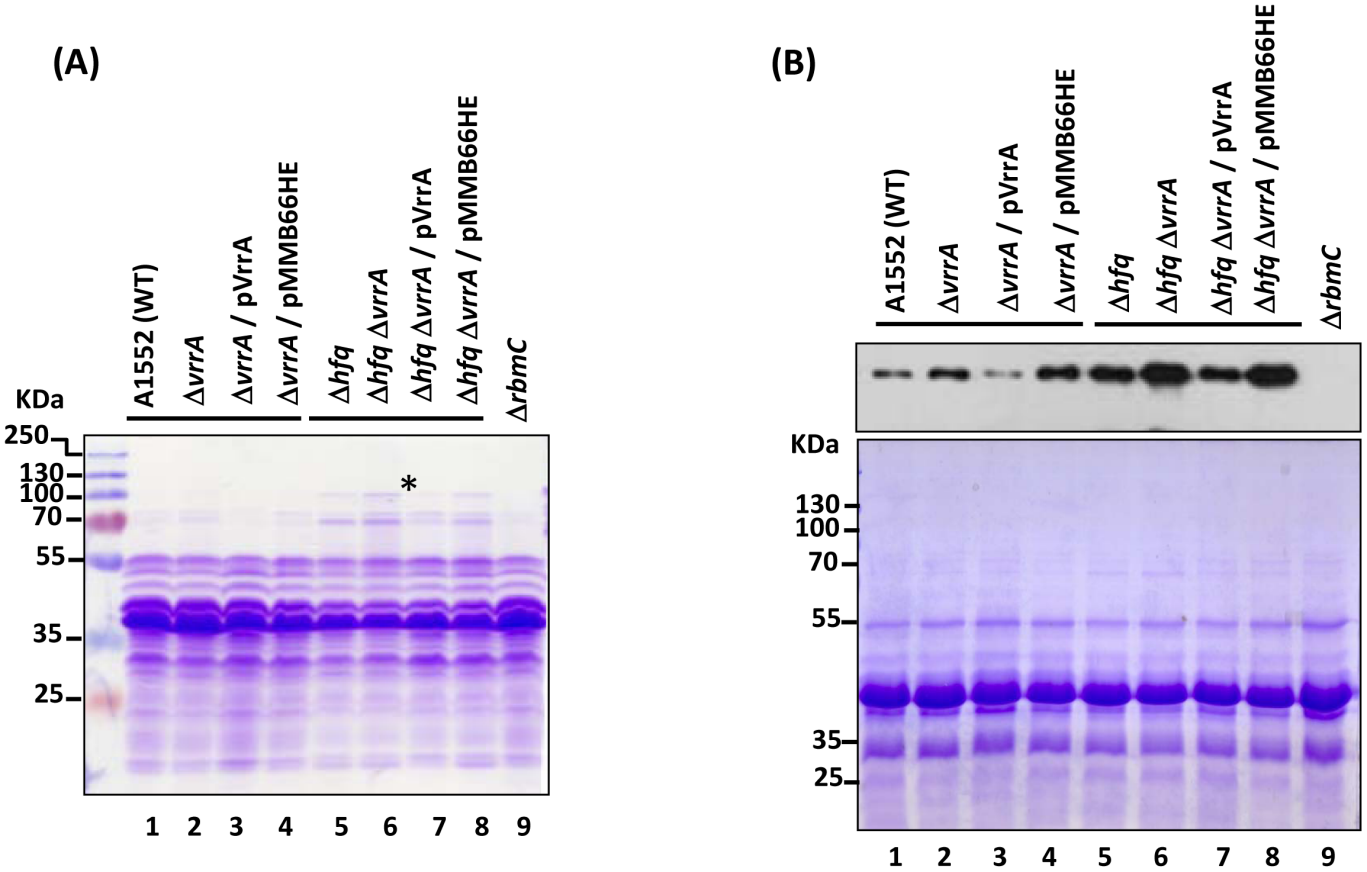
VrrA down-regulates RbmC. (A) Coomassie-brilliant-blue-stained SDS-PAGE gel. (B) Western blot detecting RbmC (upper panel); proteins on the Western blot membrane were stained with Coomassie-brilliant-blue and shown as loading control (lower panel). Supernatant samples were prepared from bacteria cultivated in LB medium at 30°C. Lane 1, A1552 (wild type); lane 2, DNY7 (Δ*vrrA*); lane 3, DNY11 (Δ*vrrA*+p*vrrA*); lane 4, DNY12 (Δ*vrrA*+pMMB66HE); lane 5, DNY8 (Δ*hfq*); lane 6, DNY9 (Δ*hfq*Δ*vrrA*); lane 7, DNY16 (Δ*hfq*Δ*vrrA*+p*vrrA*); lane 8, DNY17 (Δ*hfq*Δ*vrrA*+pMMB66HE); lane 9, DHS196 (Δ*rbmC*). LaProtein marker sizes (lane M) are given to the left in kDa. The asterisk indicates the protein band that was excised from the gel and subjected to mass spectrometry analysis.

In our previous studies, VrrA was shown to down-regulate bacterial structural proteins such as OmpA, TcpA and OmpT [35], [36]. When we analyzed the profile of secreted proteins by SDS-PAGE and Coomassie-brilliant-blue staining, we noticed that a protein band at ≈100 kDa was more abundant in the Δ*hfq* background than in the wild-type background (Fig. 1, compare lanes 5–8 to lanes 1–4). Further, this protein appeared to be more abundant in the Δ*hfq*Δ*vrrA* strain than in the Δ*hfq* strain, and the lower level was restored in the *vrrA* complemented strain (Fig. 1A, lanes 7 and 8). The protein band, marked with asterisk in Fig. 1A lane 6, was excised from the gel, subjected to mass spectrometry analysis, and identified as the biofilm matrix protein RbmC (VC0930).

In order to detect the low levels of RbmC in the wild-type background, we performed Western blot analysis using anti-RbmC polyclonal antiserum [38]. As expected, the antiserum could detect RbmC in the wild-type strain (Fig. 1B, upper panel, lanes 1–4) while no band was detected in a Δ*rbmC* mutant (Fig. 1B, upper panel, lane 9), confirming antibody specificity. Similar to what was earlier noticed in the Δ*hfq* background strains, the RbmC level was elevated in the absence of VrrA in the wild-type background strains and this elevated level was also reduced when the Δ*vrrA* strain was complemented with VrrA expressed from a plasmid (Fig. 1B, upper panel, lanes 1–4). A SDS-PAGE Coomassie blue staining gel was shown (Fig. 1B, lower panel) as a sample loading control. These data indicated that the VrrA-mediated regulation of RbmC expression did occur in the absence of Hfq. This suggests that Hfq is not essential for RbmC repression by VrrA although it is also feasible that Hfq can enhance the repression. We also observed that in the *hfq* mutant the basal RbmC protein level was higher (compare lane 1 with lane 5 in Fig. 1B, upper panel). The apparent repression by Hfq was presumably not strictly dependent on VrrA and could possibly also be mediated by some other sRNA. The higher basal level of the RbmC protein in the *hfq* mutant could also be an indirect effect through transcriptional control by a transcriptional regulator that is affected by Hfq.

### The 5′ region of *rbmC* mRNA is responsive to VrrA regulation

In order to further study the interaction between VrrA and the *rbmC* mRNA, we first determined the transcriptional start site of *rbmC* by 5′ RACE analysis. After sequencing analysis as described in Material and Methods, the *rbmC* transcriptional start site was determined to be 125 nt upstream from the AUG start codon.

**Figure 2:**
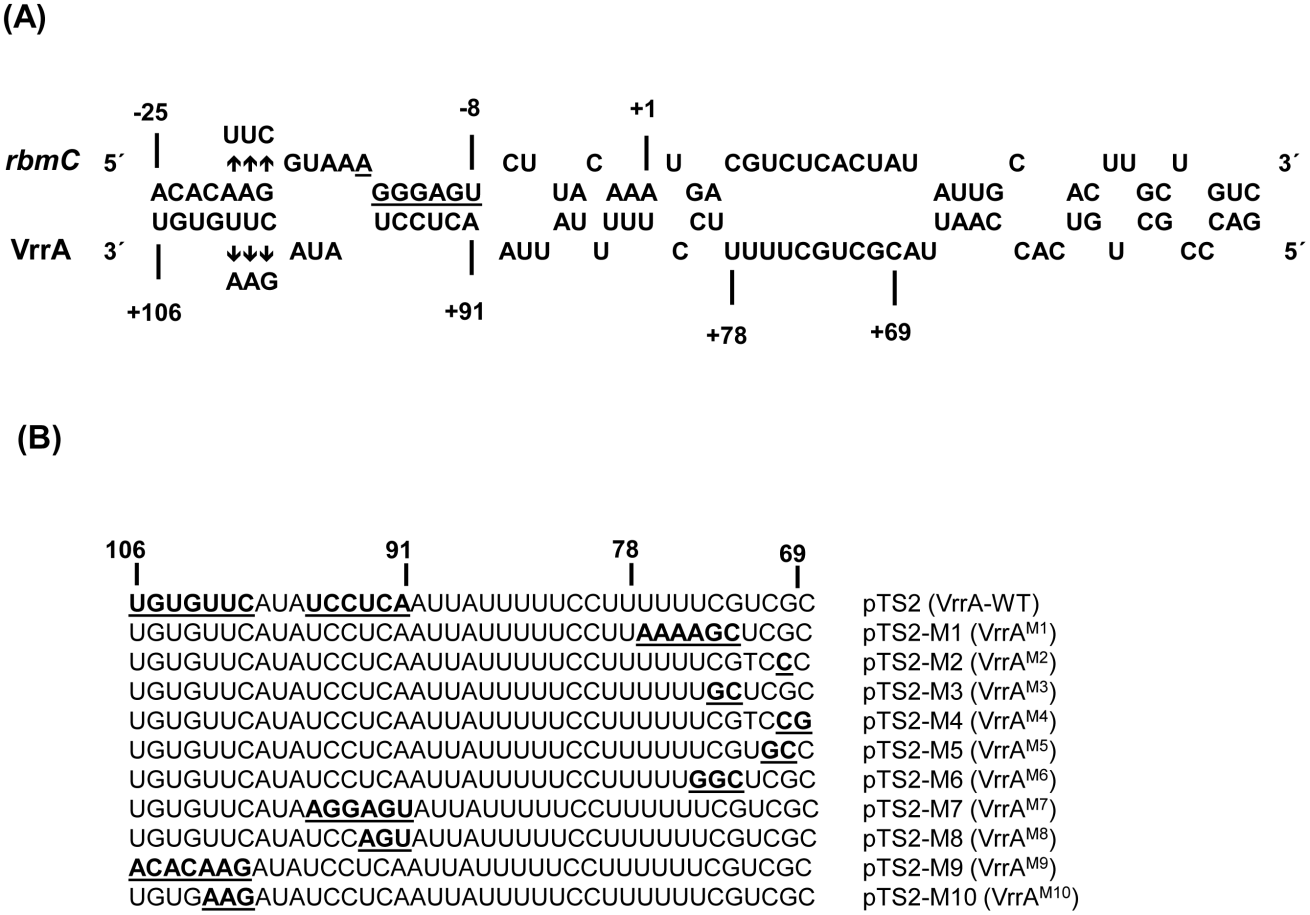
VrrA sequesters the 5′-UTR of *rbmC* by an antisense mechanism. (A) Graphical presentation of the proposed interaction of VrrA sRNA with the 5′-UTR of *rbmC* sequence, and of compensatory base-pair changes. Numbering for *rbmC* is relative to start codon AUG (A is +1), and that for VrrA is relative to the +1 transcription start site. The predicted SD sequence of *rbmC* (AGGGAGU) is underlined. Vertical arrows denote nucleotides introduced into *rbmC* and VrrA for compensatory base-pair change experiment. (B) Sequences of wild-type VrrA (pTS2) and its nucleotide substitution mutants. Nucleotides that were substituted are underlined.

Our earlier studies on the interaction between VrrA and its targets demonstrated that VrrA represses translation initiation by base-pairing with the 5′-UTR of target mRNAs (*ompA*, *tcpA* and *ompT*). We hypothesized that VrrA would interact similarly with the *rbmC* mRNA. To test this hypothesis, we used a publically available prediction program, the RNAhybrid algorithm [39], to predict possible RNA duplexes formed by VrrA and the 5′ region of the *rbmC* mRNA. The query sequence used for *rbmC* mRNA included the region from the transcriptional start site to 30 nt into the *rbmC* coding region. As shown in Fig. 2A, RNAhybrid algorithm predicted duplex formation between the residues 91–106 of VrrA and the −8 to −25 region of *rbmC* mRNA (numbering of *rbmC* is relative to the AUG start codon). This 13-bp duplex is interrupted by a bulge dividing the stretch into a 7-bp and a 6-bp duplex, with the latter masking the Shine-Dalgarno (SD) region required for translation initiation.

**Figure 3:**
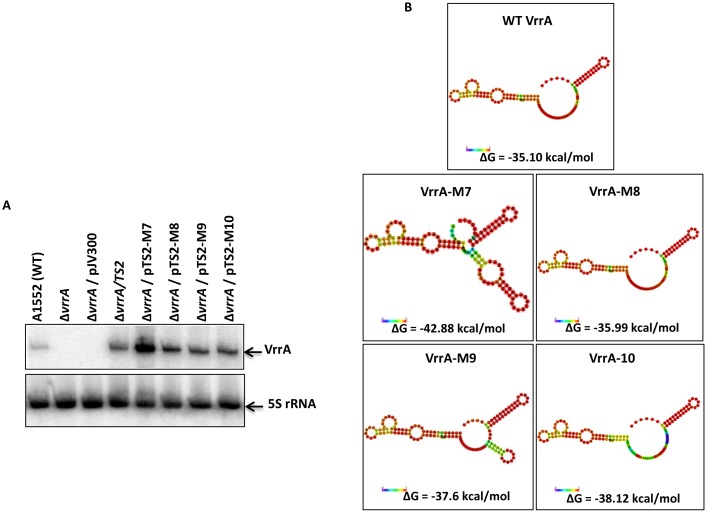
Detection of VrrA and its mutant variants by Northern blot analysis. (A) Wild-type VrrA is expressed from plasmid pTS2. Mutant variants VrrA^M7^ to VrrA^M10^ are expressed from corresponding basepair-substituted plasmids pTS2-M7 to pTS2-M10. All plasmids were transformed into *V. cholerae* strain DNY7 (Δ*vrrA*). pJV300 is used as plasmid control for pTS2. The 5S rRNA was probed as an internal control for Northern blot analysis. (B). Potential structures of VrrA variants. RNA folding was performed using the Mfold algorithm [40].

In order to dissect interacting base pairs, we introduced point mutations into VrrA (Fig. 2B). Plasmid pTS2 is a ColE1-based plasmid expressing *vrrA* from its own promoter [35]. Substitution of A_91_C_92_U_93_C_94_C_95_U_96_ with U_91_G_92_A_93_G_94_G_95_A_96_, A_91_C_92_U_93_ with U_91_G_92_A_93_, C_100_U_101_U_102_G_103_U_104_G_105_U_106_ with G_100_A_101_A_102_C_103_A_104_C_105_A_106_, and C_100_U_101_U_102_ with G_100_A_101_A_102_ generated plasmids pTS2-M7, pTS2-M8, pTS2-M9 and pTS2-M10, respectively. Each plasmid was introduced by transformation into strain DNY7 (Δ*vrrA*) and sRNA expression from the resulting plasmids were confirmed by Northern blot analysis (Fig. 3A, upper panel). The 5S rRNA was probed as internal control (Fig. 3A, lower panel). Interestingly, the VrrA-M7 level appeared higher than other VrrA variants. To compare the potential structures of these VrrA variants, RNA folding and pattern examination were performed using the Mfold web server [40]. The predicted structure of VrrA-M7 was found to be somewhat different from the predicted structures of the other variants (Fig. 3B). A feasible explanation would be that the VrrA-M7 might be more stable than wild-type VrrA, VrrA-M8, VrrA-M9, and VrrA-M10 due to a structural alteration. Another possible explanation for the higher levels of the VrrA-M7 mutant might be that this mutation could disrupt binding and co-degradation of the sRNA with another target.

**Figure 4:**
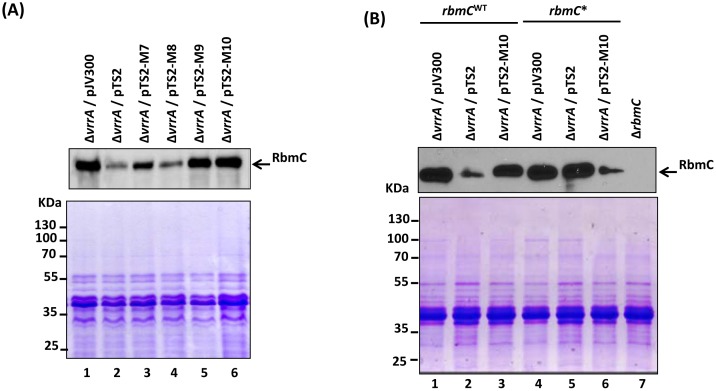
Western blot analyses of RbmC levels in the culture supernatants of the wild type *V. cholerae* strain A1552 and *vrrA* mutant derivatives: (A) Detection of RbmC (upper panel) in the supernatants from DNY7 (Δ*vrrA*) carrying different plasmids expressing either the wild-type VrrA (from plasmid pTS2) or mutant variants VrrA^M1^ to VrrA^M6^ are expressed from plasmids pTS2-M1 to pTS2-M6. A SDS-PAGE Coomassie blue stained gel is shown as the sample loading control (lower panel). (B) Western blot analysis of the RbmC levels in supernatants isolated from DNY7 (Δ*vrrA*) and DNY189 (ΔvrrA rbmC*) carrying different plasmids (upper panel). A SDS-PAGE gel stained with Coomassie blue is shown as loading (lower panel). Protein marker sizes are given to the left in kDa.

Supernatant proteins of the different sRNA-expressing strains were then analyzed to compare the production of RbmC. As shown in Fig. 4A (upper panel), compared to the wild-type VrrA^WT^ (expressed from pTS2), VrrA^M7^ (expressed from pTS2-M7) partially lost its ability to repress RbmC production whereas VrrA^M8^ (expressed from pTS2-M8) could repress RbmC production to the same extent as VrrA^WT^. In contrast, VrrA^M9^ and VrrA^M10^ (expressed from pTS2-M9 and pTS2-M10, respectively) completely lost their ability to repress RbmC production. A SDS-PAGE Coomassie blue staining gel (Fig. 4A, lower panel) was included as the sample loading control. These results show that C_100_U_101_U_102_ in VrrA are important for regulating expression of RbmC.

We next introduced mutations in the *rbmC* 5′-UTR (A_−21_A_−20_G_−19_ to U_−21_U_−20_C_−19_, Fig. 2A), generating the compensatory rbmC* allele. This rbmC* allele was introduced into the chromosome of DNY7 (Δ*vrrA*) by site-directed mutagenesis. As shown in Fig. 4B, the VrrA^M10^ variant expressed from plasmid pTS2-M10 lost its ability to repress RbmC production (Fig. 4B, upper panel, lane 3). Likewise, rbmC* was resistant to regulation by the wild-type VrrA expressed from plasmid pTS2 (Fig. 4B, upper panel, lane 5). However, regulation of rbmC* was restored upon expression of the compensatory VrrA^M10^ allele (Fig. 4B, upper panel, lane 6). A SDS-PAGE Coomassie blue stained gel was used as a sample loading control (Fig. 4B, lower panel). These data suggest that VrrA acts directly as an antisense RNA to repress *rbmC* mRNA *in vivo*.

**Figure 5:**
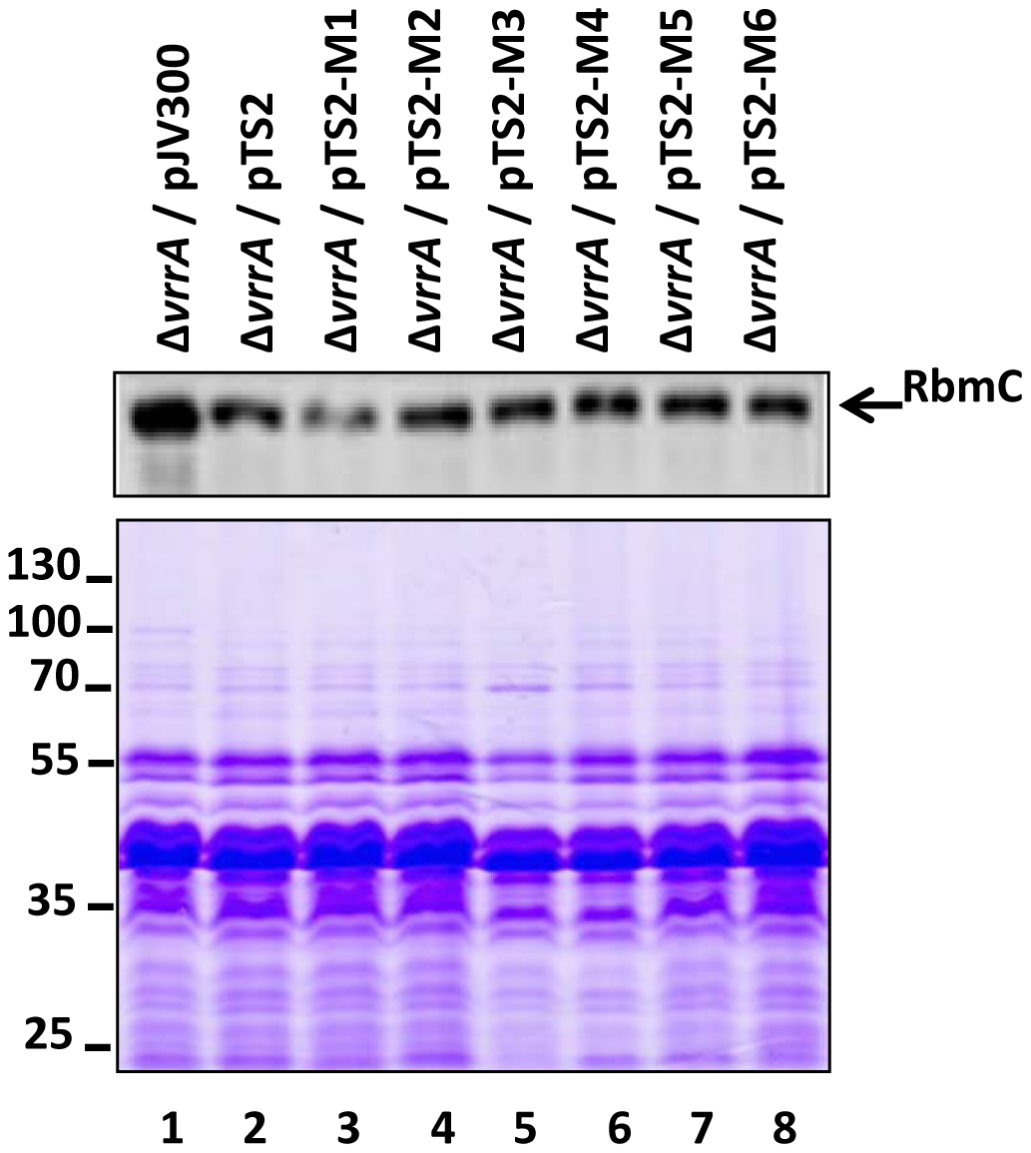
Nucleotide substitutions at residues 69–78 in VrrA do not affect repression on RbmC. Western blot analysis of supernatants from DNY7 (Δ*vrrA*) carrying the indicated plasmids (upper panel). A SDS-PAGE Coomassie blue staind gel was shown as a sample loading control (lower panel).

In our earlier study, VrrA mutant variants (VrrA^M1^ to VrrA^M6^) expressed from plasmids pTS2-M1 to pTS2-M6 (Fig. 2B) were constructed to study the interaction between VrrA and the *ompT* mRNA. We showed that VrrA mutant variants covering the VrrA region from residues 69–78 was responsible to base-pair with 5′ UTR of *ompT* mRNA [36]. In order to see whether these residues would be important for RbmC regulation as well since the residues 69–78 were closed to the interacting region, we monitored RbmC levels in the strains expressing VrrA^M1^ to VrrA^M6^ by Westen blot analysis. As shown in Fig. 5 (upper panel) none of these variants lost its ability to repress RbmC, suggesting that *ompT*- and *rbmC*-regulating regions in VrrA do not overlap.

### VrrA modulates biofilm formation

**Figure 6:**
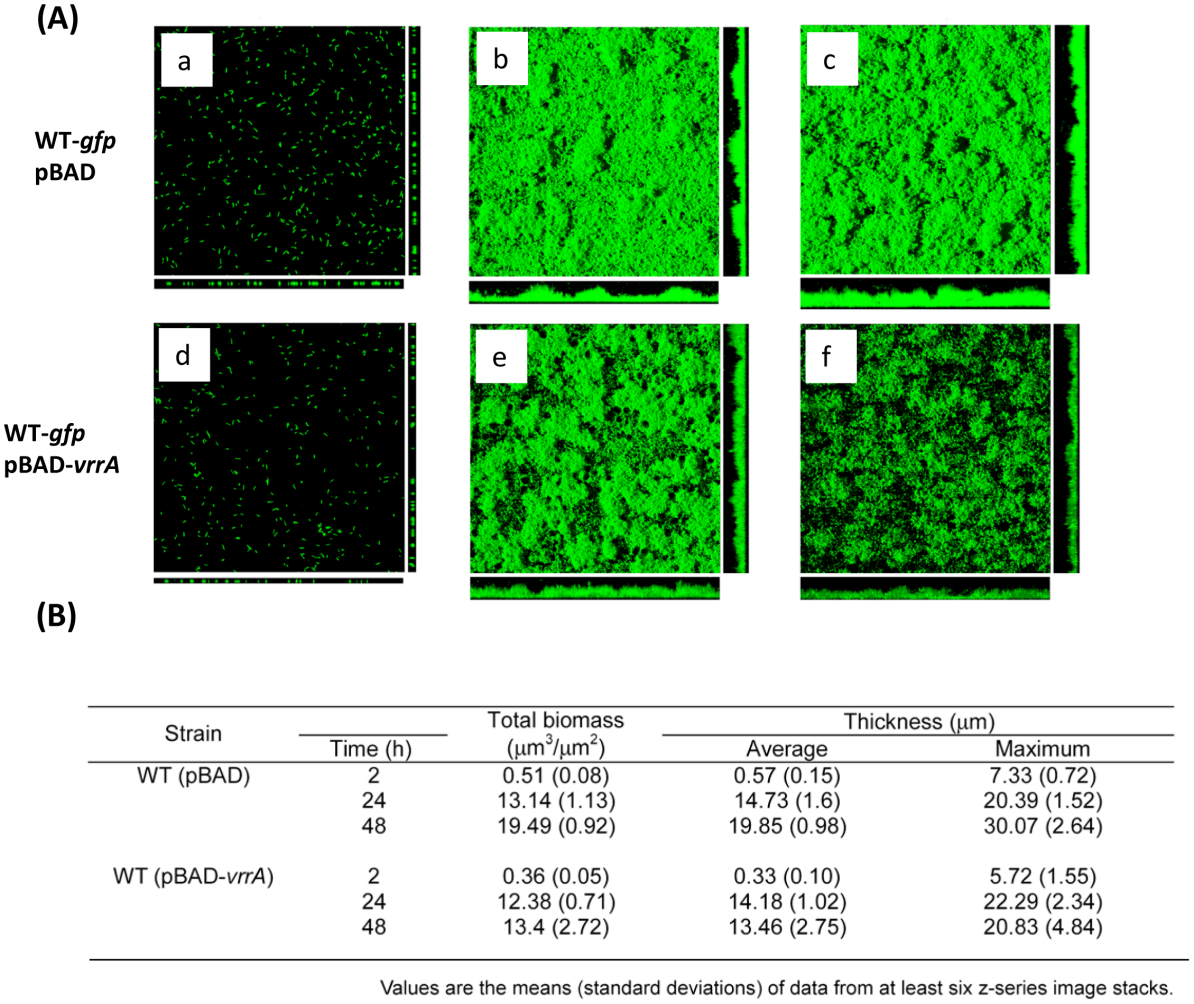
Impact of *vrrA* on biofilm formation (A) Confocal laser scanning microscopy images of horizontal (xy) and vertical (xz) projections of biofilm structures formed by wild-type strain (WT) carrying the vector or pBAD-*vrrA*. Cells were grown for 2(a and d), 24 h (b and e); 48 h (c and f) in 2% LB medium in the presence of ampicillin and 0.05% arabinose at room temperature. (B) COMSTAT analysis of biofilms formed by wild-type strains harboring a plasmid vector or *vrrA* over-expression plasmid.

The findings about the ability of VrrA to down-regulate RbmC levels prompted us to analyze the impact of VrrA on biofilm formation by *V. cholerae*. We compared the biofilm forming ability using a once-through flow cell system and analysis by confocal laser scanning microscopy (CLSM). The over-expression of VrrA from a plasmid clone in the wild-type strain markedly decreased biofilm formation at 48 h when compared to that of the same strain containing the plasmid vector (Fig. 6A, c and f). Although initial stages of biofilm formation at 2 h and 24 h were not markedly altered by *vrrA* gene overexpression (Fig. 6A, a and d; b and e), COMSTAT analysis of biofilms developed 48 h post inoculation revealed that total biomass, average and maximum thicknesses of the wild-type strain overexpressing *vrrA* were markedly decreased after 48 h compared to those of the wild-type strain harboring only the plasmid vector after 48 h although the growth rate and yield were similar between control and over-expression strains. These results show that over-expressing VrrA impairs the ability of *V. cholerae* to form biofilms.

## Discussion

*V. cholerae* transits between fundamentally different habitats the aquatic environment and the human digestive tract. Such transitions require rapid acquisition and integration of environmental cues in order to coordinate adequate genetic programs and adapt to the new niche. One such adaptation program involves the switch between a planktonic, motile lifestyle and a biofilm-based sessile lifestyle. To date, numerous regulator proteins have been found to affect biofilm formation in *V. cholerae*, such as those described in the Introduction. Results from this study add a new class of regulators, sRNAs, as a direct regulator of a biofilm matrix component. Through down-regulation of RbmC, VrrA weakens the stability of the mature biofilm structure and might therefore facilitate dispersal of bacteria from a sessile to a planktonic life style. In addition, because expression of VrrA is controlled by sigma factor σ^E^, VrrA serves as a molecular link between the σ^E^-regulon and biofilm formation in *V. cholerae*.

Several sRNAs have been shown to be involved in biofilm formation in *E. coli* and *Salmonella*, e. g. OmrA/B [41], McaS [42], [43], RprA [44] and GcvB [42]. In contrast to VrrA, these sRNAs do not target biofilm matrix components directly, instead they target biofilm master regulators such as CsgD, which in turn regulates biofilm components. This generates a hierarchical regulatory network and enables *csgD* mRNA to serve as a hub for complex signal integration via multiple sRNAs [45], [46]. Similarly in *Vibrio*, sRNAs Qrr1-4 and CsrB/C/D regulate the biofilm master regulator HapR or the regulatory molecule cyclic di-GMP (through diguanylate cyclase) [47], [48], and thus are indirectly involved in biofilm formation.

VrrA belongs to a growing family of sRNAs that regulate multiple targets [48], [49]. VrrA uses unique pairing regions to differentially regulate different mRNA targets. Compensatory base pair change experiments revealed that residues C_100_U_101_U_102_ (numbers relative to the +1 transcriptional start site) in VrrA are essential for base-pairing with *rbmC* mRNA, while those required for the regulation of *ompT* mRNA are G_73_C_74_U_75_ in VrrA [36].

In addition to the target-specific regulating regions in VrrA, dependency on the chaperon protein Hfq differs among mRNA targets as well. Although deletion of *hfq* abolishes the interaction between VrrA and *ompT* mRNA, Hfq is not absolutely required for the regulation on *ompA* [35] or *rbmC* mRNAs (this study). The observation that OmpA and RbmC levels were elevated in the Δ*hfq* strain and that VrrA could only partially repress this elevated expression suggests that additional sRNAs are involved in the regulation. The combination of target-specific regions in VrrA and differentiated requirement of Hfq allows VrrA to modulate multiple targets differentially.

According to the RNAhybrid prediction, as shown in Fig. 2, A_91_C_92_U_93_C_94_C_95_U_96_ in VrrA base pairs to the potential SD sequence (AGGGAGU) of *rbmC*. We therefore expected to see the most drastic change in RbmC level in strains expressing VrrA^M7^ (substitution of A_91_C_92_U_93_C_94_C_95_U_96_ with U_91_G_92_A_93_G_94_G_95_A_96_) and VrrA^M8^ (substitution of A_91_C_92_U_93_ with U_91_G_92_A_93_). However, our results showed that VrrA^M9^ and VrrA^M10^, which base pairs to the region upstream of the SD sequence, had more impact on the regulation of RbmC. This unexpected result might be due to the fact that the SD sequence was predicted based on the consensus sequence and therefore might not be the exact SD site. Future studies using e. g. toeprint analyses will hopefully identify the actual interaction site(s) between VrrA and *rbmC* mRNA. Nevertheless, the present results from the compensatory base pair substitution experiment demonstrate that there is a direct interaction between VrrA and *rbmC* at the region upstream of the putative SD sequence (Fig. 4B).

It is noteworthy that there are only a few functional homologs to VrrA in other Gram-negative bacteria. One such example is the MicA sRNA in *Salmonella* and *E. coli* [50], [51]. Both MicA and VrrA are σ^E^-dependent and are capable of down-regulating multiple outer membrane proteins by base-pairing mechanisms [35], [52]. Interestingly, Kint et al [45] observed that MicA in *Salmonella* was involved in biofilm formation, although the molecular mechanism remains unknown. Systematic searches for MicA targets using bioinformatics prediction tools have not identified yet any biofilm-related genes. Future work will be needed to examine possible interactions between MicA and *Salmonella* biofilm components such as curli and fimbriae.

In summary, VrrA is the first example of an sRNA molecule that directly targets expression of a biofilm matrix component. Given the similarities between VrrA and its homologs in other Gram-negative bacteria, it is plausible that similar direct regulation exists in other bacteria as well. Because VrrA weakens the stability of the mature biofilm structure, strategies directed towards mechanisms or levels of sRNAs to disturb bacterial biofilm formation may potentially be used to combat biofilm-related infections. Furthermore, in our earlier studies, we showed that the TcpA, one of the colonization factors of *V. cholerae*, was down-regulated by VrrA (Song et al. 2008). In this study, we demonstrated that the expression of one of the extracellular matrix proteins, RbmC that is important for the biofilm formation by *V. cholerae* was modulated by VrrA. We hypothesize that at the later stage of *V. cholerae* infection in the host, bacteria can move away from the epithelial surface and into the fluid-filled lumen of the intestine. During this time, the bacteria may undergo a switch from attachment to the epithelial surface to detachment. This process may be associated with up-regulation of VrrA. We suggest that this transition prepares the bacteria to leave the intestine, for survival in the environment, and for eventual transmission to a new host. This process might be orchestrated by VrrA that can modulate expression of both a colonization factor (Tcp) and attachment factor (RbmC).

## Materials and Methods

### Oligonucleotides

The complete list of DNA oligonucleotides used for cloning and generating probes in hybridization is provided in Table 1.

**Table 1:** Oligonucleotides used in this study.

Primer	Sequence in 5′→3′ direction	Restriction site	Used for construction of
DS-5	CCGAATTCCACATTTTCTGCCATGTCTG	*EcoR1*	pBAD18/*vrrA*
DS-6	CCTCTAGAGCCAATGAACCGACTTGAAC	*Xba1*	pBAD18/*vrrA*
DS-67	CGCTCTAGACTCCTGTAGGGATAATTAAGGC	*XbaI*	Δ*rbmC*
DS-68	CCCATCCACTAAACTTAAACAAGACGTCATTTGTAAGACTCC		Δ*rbmC*
DS-69	TGTTTAAGTTTAGTGGATGGGGTCTACTAACGACTCATCGCT		Δ*rbmC*
DS-70	CGCTCTAGACTCTTACAATCAAGGCGAAG	*XbaI*	Δ*rbmC*
TIS-94	CGCTCTAGAATATGTAACGCAAGATGCCAC	*XbaI*	rbmC*
TIS-95	CGCTCTAGAATCCCAATCACTTAGCATGAC	*XbaI*	rbmC*
TIS-96	TTAACACAAGCTAAAGGGAGTCTTACAAATGA	*AluI*	rbmC*
TIS-97	GACTCCCTTTAGCTTGTGTTAATTTTATTCAA	*AluI*	rbmC*
TIS-98	TTAACACTTCGTAAAGGGAGTCTTACAAATGA		rbmC*
TIS-99	GACTCCCTTTACGAAGTGTTAATTTTATTCAA		rbmC*
TIS-79	GTTTTTGCTAGCACTCAACGACAAAAGACCGAC		5′ RACE
TIS-86	CTTTTTATTATGAGGAATACTTGTGTACGCCCAAAGC		pTS2-M7
TIS-87	TACACAAGTATTCCTCATAATAAAAAGGAAAAAGCAG		pTS2-M7
TIS-88	CCTTTTTATTATGACCTATACTTGTGTACGCCCAAAGC		pTS2-M8
TIS-89	TACACAAGTATAGGTCATAATAAAAAGGAAAAAGCAGC		pTS2-M8
TIS-90	CTATAGAACACAACGCCCAAAGCCAGATTG		pTS2-M9
TIS-91	TTGGGCGTTGTGTTCTATAGGAGTTAATAA		pTS2-M9
TIS-92	CCTATAGAAGTGTACGCCCAAAGCCAGATTG		pTS2-M10
TIS-93	TTGGGCGTACACTTCTATAGGAGTTAATAAA		pTS2-M10
JVO-8106	CTGTTTCGTTTCACTTCTGAGTTC		5S rRNA probe
JVO-8109	AACCAAATTTGACGGCCAGT		VrrA probe

### Bacterial strains and growth conditions

Strains used in this study are listed in Table 2. *V. cholerae* El Tor Inaba strain A1552 is referred to as the wild-type throughout this study. *V. cholerae* strains were grown in LB at 37°C or 30°C, as indicated. Carbenicillin was supplemented at 100 µg ml^−1^ when appropriate.

**Table 2:** Bacterial strains and plasmids used in this study.

Strain or plasmid	Description or relevant genotype	Source or reference
***V. cholerae*** ** strains**		
A1552	Wild-type *V. cholerae* El Tor Inaba	[59]
DNY7	A1552Δ*vrrA*	[35]
DNY8	A1552Δ*hfq*	[35]
DNY9	A1552Δ*vrrA*Δ*hfq*	[35]
DNY11	A1552Δ*vrrA*+p*vrrA*	[35]
DNY12	A1552Δ*vrrA*+pMMB66HE	[35]
DNY16	A1552Δ*vrrA*Δ*hfq*+p*vrrA*	[35]
DNY17	A1552Δ*vrrA*Δ*hfq*+pMMB66HE	[35]
DHS196	A1552Δ*rbmC*	This study
DHS284	A1552Δ*bap1*	[38]
DNY188	A1552Δ*vrrA rbmC*-intermediate	This study
DNY189	A1552ΔvrrA rbmC*	This study
DNY34	A1552Δ*vrrA*+pJV300	[36]
DNY35	A1552Δ*vrrA*+pTS2	[36]
DNY44	A1552Δ*vrrA*+pTS2-M1	[36]
DNY63	A1552Δ*vrrA*+pTS2-M2	[36]
DNY64	A1552Δ*vrrA*+pTS2-M3	[36]
DNY65	A1552Δ*vrrA*+pTS2-M4	[36]
DNY66	A1552Δ*vrrA*+pTS2-M5	[36]
DNY156	A1552Δ*vrrA*+pTS2-M6	[36]
DNY178	A1552Δ*vrrA*+pTS2-M7	This study
DNY179	A1552Δ*vrrA*+pTS2-M8	This study
DNY180	A1552Δ*vrrA*+pTS2-M9	This study
DNY181	A1552Δ*vrrA*+pTS2-M10	This study
DHS420	A1552ΔvrrA rbmC*+pJV300	This study
DHS422	A1552ΔvrrA rbmC*+pTS2	This study
DHS424	A1552ΔvrrA rbmC*+pTS2-M10	This study
WT-*gfp*	A1552-*gfp*	This study
WT-*gfp*/pBAD	A1552-*gfp*/pBAD	This study
WT-*gfp*/p*vrrA*	A1552-*gfp*/p*vrrA*	This study
**Plasmids**		
pMMB66HE	Control plasmid	[60]
pBAD18	Control plasmid	[54]
pBAD18/*vrrA*	*vrrA* complementation plasmid, based on pBAD18	This study
p*vrrA*	*vrrA* complementation plasmid, based on pMMB66HE	[35]
pJV300	ColE1 plasmid expressing a ≈50-nt nonsense transcript	[55]
pTS2	ColE1 plasmid expressing *vrrA* from its own promoter	[35]
pTS2-M1	pTS2 carrying a 6-nt substitution in putative *ompT* interaction sequence, as shown in Fig. 5A	[36]
pTS2-M2	pTS2 carrying a single-nucleotide substitution in putative *ompT* interaction sequence, as shown in Fig. 5A	[36]
pTS2-M3	pTS2 carrying a 2-nt substitution in putative *ompT* interaction sequence, as shown in Fig. 5A	[36]
pTS2-M4	pTS2 carrying a 2-nt substitution in putative *ompT* interaction sequence, as shown in Fig. 5A	[36]
pTS2-M5	pTS2 carrying a 2-nt substitution in putative *ompT* interaction sequence, as shown in Fig. 5A	[36]
pTS2-M6	pTS2 carrying a 3-nt substitution in putative *ompT* interaction sequence, as shown in Fig. 5A	[36]
pTS2-M7	pTS2 carrying a 6-nt substitution in putative *rbmC* interaction sequence, as shown in Fig. 2B	This study
pTS2-M8	pTS2 carrying a 3-nt substitution in putative *rbmC* interaction sequence, as shown in Fig. 2B	This study
pTS2-M9	pTS2 carrying a 6-nt substitution in putative *rbmC* interaction sequence, as shown in Fig. 2B	This study
pTS2-M10	pTS2 carrying a 3-nt substitution in putative *rbmC* interaction sequence, as shown in Fig. 2B	This study

### DNA manipulations

An in-frame deletion of *rbmC* in A1552 resulting in strain DHS196 was performed using the method described by Skorupski and Taylor [53]. Primer sequences are summarized in Table 1. The rbmC* allele was introduced into the chromosome of DNY7 (Δ*vrrA*) by site-directed mutagenesis, resulting in strain DNY189. The site-directed mutagenesis experiment was performed as previously described [36], with the addition of an intermediate step using strain DNY188. Primers TIS-96 and TIS-97 were used to introduce a nucleotide change (from −21AAGGT to −21AAGCT) into DNY7, resulting in strain DNY188; primers TIS98 and TIS-99 were used to introduce nucleotide changes (from −21AAGCT to −21TTCGT) into DNY188, resulting in strain DNY189. The intermediate strain DNY188 contains an AluI restriction site (AGCT), which allows for mutant screening. Generation of GFP-tagged *V. cholerae* wild-type strain A1552 was performed as described in the earlier studies [16]. A DNA fragment (304 bp) containing the *vrrA* gene including its putative promoter region was amplified from the A1552 genome and cloned into pBAD18 vector [54]at the EcoR1/Xba1 sites. The resulting plasmid pBAD/*vrrA* and its vector control (pBAD18) were introduced by transformation into the wild type *V. cholerae* strain A1552-*gfp*, resulting WT-*gfp*/pBAD and WT-*gfp*/pBAD-*vrrA* respectively.

Plasmid pTS2 is a ColE1-based plasmid expressing wild-type VrrA from its own promoter [35]. This plasmid served as template for the construction of plasmids pTS2-M7, pTS2-M8, pTS2-M9 and pTS2-M10 that carry the nucleotide changes shown in Fig. 2B. Procedures were performed as described earlier [55], and primers used to introduce nucleotide change are summarized in Table 2.

### SDS-PAGE and Western blot analysis

Protein samples were prepared from equal amounts of bacteria cells after overnight growth at 30°C. Bacteria were harvested by centrifugation at 10,000×g for 10 min at 4°C. The culture supernatant fluid was precipitated with 10% trichloroacetic acid (TCA). Briefly, 1 volume (250 µl) of 50% TCA stock was added to 4 volumes (1 ml) of protein sample. The protein-TCA mixture was kept on ice for 15 min, and subsequently the tube was centrifuged at 15,000×g for 5 min. The supernatant was removed and the protein pellet was washed with 200 µl of cold acetone. Finally, the tube was centrifuged at 15,000×g for 5 min and the resulting pellet was dissolved in sample buffer containing 10% glycerol, 0.05% bromophenol blue, 2% SDS, 5% 2-mercaptoethanol, and 10 mM Tris-HCl, pH 6.8. Proteins with known molecular masses (Fermentas) were used as molecular mass markers. SDS-PAGE and Western blotting were carried out according to the methods of Laemmli [56] and Towbin et al. [57]. HRP-conjugated donkey anti-rabbit IgG (Promega, USA) was used as secondary antibody. Detection was performed using ECL Prime Western Blotting Detection Reagent (Amersham or GE Life Sciences, USA). Pre-stained Protein Ladder (SM0679, Fermentas) was used as size standards. Gels were stained with Coomassie brilliant blue.

### RNA isolation and Northern blot analysis

RNA samples were prepared as previously described [36] from bacterial cultures grown overnight (14 hr) at 37°C. The RNA was treated with DNase I and quantified on a NanoDrop ND-1000 Spectrophotometer (NanoDrop Technologies, USA). For Northern blot analysis, 10 µg RNA sample was resolved in a polyacrylamide gel and transferred to a Hybond-XL membrane (GE Healthcare, USA) by electro-blotting (1 h, 50 V, 4°C) in a tank blotter. Radiolabeled probes were used to visualize the required mRNA or sRNA. Northern blots were exposed to a phosphorimager screen and scanned on a Storm™ phosphorimager (Molecular Dynamics, USA). Quantification was performed using Quantity One software (Roche, USA). For VrrA and 5S rRNA detection, radio labeled (γ-P32-ATP) oligo probe JVO-8109 and JVO-8106 was used respectively.

### 5′ RACE analysis

5′ RACE was performed as previously described [55] to determine the transcription start site of *rbmC*. Total RNA isolated from the wild-type *V. cholerae* strain A1552 was used to generate cDNA. Oligo TIS-79 (Table 1) was used as *rbmC*-specific primer in PCR. PCR products were separated on a 2% agarose gel, gel-eluted and used as template for sequencing.

### Mass spectrometry peptide sequencing

Proteins of interest were excised from the Coomassie-stained SDS-PAGE gel and analyzed by Alphalyse (Denmark) for mass spectrometry.

### Biofilm analysis

Flow cell experiments were carried out according to the procedure previously described [58]. Briefly, overnight-grown cultures of *gfp*-tagged *V. cholerae* strains were diluted to an optical density at 600 nm (OD_600_) of 0.02 in 2% LB (0.02% tryptone, 0.01% yeast extract, 1% NaCl; pH 7.5) containing 100 µg/ml of ampicillin and used to inoculated flow chambers. Flow cell experiments were carried out at room temperature with 2% LB containing ampicillin (100 µg/ml) and arabinose (0.2%, wt/vol). CLSM images of the biofilms were captured with a LSM 5 PASCAL system (Zeiss) at 488 nm excitation and 543 nm emission wavelengths. Three dimensional images of the biofilms were reconstructed using Imaris software (Bitplane) and quantified using COMSTAT (Heydorn and Molin, 2000). Flow cell experiments were carried out with at least two biological replicates.
